# Digitally Disconnected: Qualitative Study of Patient Perspectives on the Digital Divide and Potential Solutions

**DOI:** 10.2196/33364

**Published:** 2021-12-15

**Authors:** Maria Alcocer Alkureishi, Zi-Yi Choo, Ali Rahman, Kimberly Ho, Jonah Benning-Shorb, Gena Lenti, Itzel Velázquez Sánchez, Mengqi Zhu, Sachin D Shah, Wei Wei Lee

**Affiliations:** 1 Department of Pediatrics University of Chicago Chicago, IL United States; 2 Pritzker School of Medicine University of Chicago Chicago, IL United States; 3 University of Chicago Chicago, IL United States; 4 New York University Long Island School of Medicine Mineola, NY United States; 5 Department of Internal Medicine University of Washington Seattle, WA United States; 6 Department of Medicine University of Chicago Chicago, IL United States

**Keywords:** telemedicine, digital divide, patient experience, qualitative study

## Abstract

**Background:**

As telemedicine utilization increased during the COVID-19 pandemic, divergent usage patterns for video and audio-only telephone visits emerged. Older, low-income, minority, and non-English speaking Medicaid patients are at highest risk of experiencing technology access and digital literacy barriers. This raises concern for disparities in health care access and widening of the “digital divide,” the separation of those with technological access and knowledge and those without. While studies demonstrate correlation between racial and socioeconomic demographics and technological access and ability, individual patients’ perspectives of the divide and its impacts remain unclear.

**Objective:**

We aimed to interview patients to understand their perspectives on (1) the definition, causes, and impact of the digital divide; (2) whose responsibility it is to address this divide, and (3) potential solutions to mitigate the digital divide.

**Methods:**

Between December 2020 and March 2021, we conducted 54 semistructured telephone interviews with adult patients and parents of pediatric patients who had virtual visits (phone, video, or both) between March and September 2020 at the University of Chicago Medical Center (UCMC) primary care clinics. A grounded theory approach was used to analyze interview data.

**Results:**

Patients were keenly aware of the digital divide and described impacts beyond health care, including employment, education, community and social contexts, and personal economic stability. Patients described that individuals, government, libraries, schools, health care organizations, and even private businesses all shared the responsibility to address the divide. Proposed solutions to address the divide included conducting community technology needs assessments and improving technology access, literacy training, and resource awareness. Recognizing that some individuals will never cross the divide, patients also emphasized continued support of low-tech communication methods and health care delivery to prevent widening of the digital divide. Furthermore, patients viewed technology access and literacy as drivers of the social determinants of health (SDOH), profoundly influencing how SDOH function to worsen or improve health disparities.

**Conclusions:**

Patient perspectives provide valuable insight into the digital divide and can inform solutions to mitigate health and resulting societal inequities. Future work is needed to understand the digital needs of disconnected individuals and communities. As clinical care and delivery continue to integrate telehealth, studies are needed to explore whether having a video or audio-only phone visit results in different patient outcomes and utilization. Advocacy efforts to disseminate public and private resources can also expand device and broadband internet access, improve technology literacy, and increase funding to support both high- and low-tech forms of health care delivery for the disconnected.

## Introduction

Prior to the COVID-19 pandemic, the use of synchronous telemedicine (eg, audio-only telephone or video visits) in the United States was limited and mainly incorporated in specialty fields, such as postoperative care and psychiatry [1,2]. In response to the pandemic, the Centers for Medicare and Medicaid Services (CMS) and private insurers expanded coverage for both video and phone telehealth visits, and telemedicine utilization increased exponentially [3-5] and expanded into primary care [6,7]. While further study is needed to explore the challenges of telemedicine [8], initial studies have found that with certain patient populations and conditions, telemedicine is associated with a number of patient and clinician benefits, including reduced appointment wait times, costs, improved medication adherence and blood pressure control, and high rates of patient and clinician satisfaction [9-19].

However, as telemedicine utilization increased, diverging usage patterns for video and audio-only telephone visits emerged, raising concerns about the widening “digital divide” contributing to disparities in telehealth access [7,20-22]. The “digital divide” refers to a societal division between those who have the technological means to make full use of technology and those who face barriers preventing proper use and benefit [20]. Access to high-speed internet and technology devices (eg, computers, tablets, smartphones) and a degree of digital literacy are required to successfully participate in video visits [7,23]. As health care becomes more reliant on technology-based tools, the digital divide stands to further exacerbate existing health care access disparities.

Studies have shown that patients with lower levels of digital literacy and access to technology are more likely to be from marginalized backgrounds, including older, Black and Hispanic, non-English speaking patients, and those with Medicaid insurance [24]. Further, during the pandemic, patients from disadvantaged socioeconomic backgrounds were less likely to complete video visits and more likely to rely on audio-only telephone visits to access their providers and telehealth utilization data from federally qualified health centers confirmed these findings [7,20,25-28]. Because a third of Medicare telehealth encounters were audio-only phone visits between March and June 2020, significant concerns around worsening health inequalities are raised if reimbursement parity between video and telephone visits is discontinued in the future [4,23,29-31].

While studies have assessed the demographics of the digital divide, none have directly explored patient perspectives on the digital divide and potential impacts. We aimed to interview patients to understand their perspectives on (1) the definition, causes, and impact of the digital divide; (2) whose responsibility it is to address the digital divide; and (3) potential solutions to mitigate the digital divide.

## Methods

### Setting

The UCMC serves a diverse medically underserved patient population on the South Side of Chicago. During the COVID-19 pandemic, UCMC began offering virtual visits in March 2020 in response to the March 6, 2020, policy changes and regulatory waivers from CMS and provisions of the US Coronavirus Aid, Relief, and Economic Security Act, effective March 27, 2020 [3,7]. From December 2020 to March 2021, we conducted semistructured telephone interviews with adult patients and parents of pediatric patients who had virtual visits (phone, video, or both) at UCMC adult or pediatric primary care clinics from March 2020 to September 2020.

### Researcher Characteristics

The research team consisted of 2 faculty physicians (MA and WL), 2 medical students (GL and Z-YC), and 4 undergraduate research assistants (AR, IVS, JB-S, and KH). Interviews were conducted by AR, GL, IVS, JB-S, KH, and Z-YC. Qualitative analysis was performed by AR, JB-S, MA, WL, and Z-YC.

### Interview Guide Development

The interview script was part of a larger qualitative interview study focused on understanding patients’ overall telehealth experiences during the pandemic. The second half of the interview focused on digital divide perspectives, and the script was developed after a literature review on the digital divide in health care and patient perspectives on telehealth. Results from an internal Press Ganey patient telemedicine survey were used to guide question development. An advisory group of key institutional leadership and stakeholders at UCMC (eg, Vice President and Chief Ambulatory Medical Officer, Associate Chief Medical Information Officer, Advancement Manager for Health Literacy, Diversity and Inclusion) provided feedback on the interview guide and patient recruitment. Patients and family members from the UCMC Ambulatory Patient and Family Advisory Council also provided feedback.

The interview guide (Multimedia Appendix 1) included 4 demographic questions and 6 open-ended questions to elicit perspectives on how patients define the digital divide, its impacts, who they believe is responsible for addressing the divide, and how it could be resolved. A definition of the digital divide was provided to all participants, regardless of their ability to correctly define the concept or not. All research assistants completed pilot interviews, received feedback from the senior authors MA and WL, and revisions were made to the interview script to improve question clarity and focus.

### Sampling Strategy

Details on all adult and pediatric primary care patients who had phone, video, or both virtual visit types at UCMC between March 2020 and September 2020 were extracted from the electronic health record and these patients were eligible for inclusion in the study. Patient demographic data were also obtained (eg, visit type/date, insurance type, primary language, age, sex, race, and phone number).

We purposefully sampled patients to ensure we captured patient experiences for both adult and pediatric patients who had different visit types (phone, video, or both). Patients were randomly chosen until we had representative participants from each subgroup (adult phone, adult video, adult both, pediatric phone, pediatric video, and pediatric both). Participants received up to 2 phone calls to invite them to participate, and oral consent was obtained. We aimed to complete between 25 and 50 total interviews based on prior qualitative studies in our patient population and previous telehealth studies [32-37]. Patient recruitment continued until thematic saturation was reached. All participants received a US $20 gift card to compensate them for their time.

### Data Analysis

A total of 54 phone interviews were conducted, digitally recorded, assigned a randomized subject identification number, and submitted for professional transcription. Three research assistants (GL, IVS, and KH) reviewed and deidentified transcripts to ensure accuracy and anonymity. ATLAS.ti 9 was used for qualitative analysis [38]. Using a constant comparative approach, a coding team (AR, MA, JB, WL, and ZC) performed iterative content analysis of 3 transcripts. An additional 9 interviews were reviewed independently and discussed as a group until the code book was finalized and consensus was reached on theme saturation. The remaining 42 interviews were analyzed by AR, JB, and ZC. All coded transcripts were validated by at least one of two reviewers (MA and WL). Both reviewers also independently coded a subset of the interviews, comparing analyses to ensure effective data triangulation.

### Institutional Approval

The project conforms with the Standards for Reporting Qualitative Research [39], and was approved as a quality improvement project by the University of Chicago. As such, this initiative was deemed not human subjects research and was not reviewed by the Institutional Review Board.

## Results

### Overview

**Figure 1:**
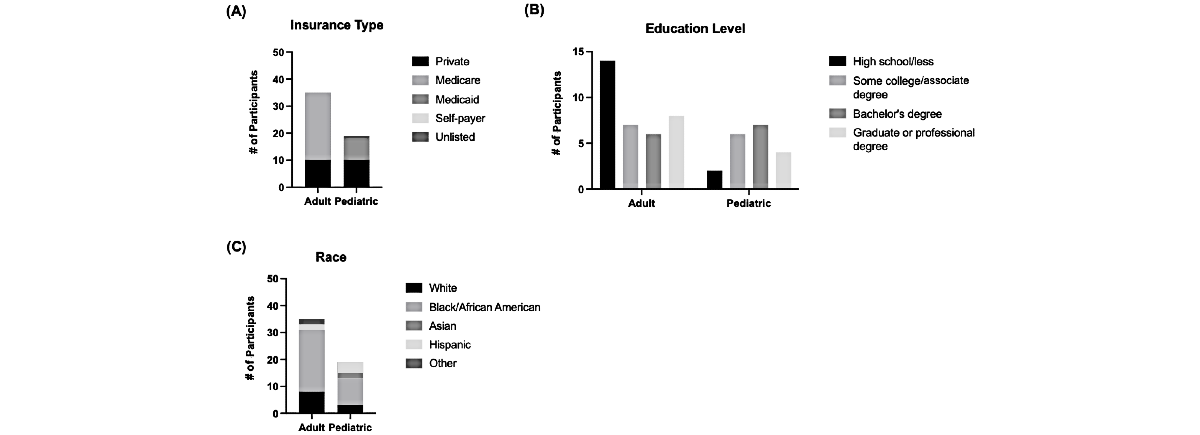
Respondent demographics by (A) insurance, (B) highest level of educational attainment and (C) race.

A total of 216 adult patients were contacted for the study, and 35 consented and completed interviews. In pediatrics, 104 parents were contacted, and 19 consented and completed the interviews. The majority of respondents were adult primary care patients (35/54, 65%), and 35% (19/54) were parents of pediatric patients. Interviews lasted an average of 42 minutes (range 23-79 minutes). Most participants were female (35/54, 65%) and the average age was 55 years (Table 1). The only significant difference between adult and pediatric parent participants was their age (average age: adults: 63.6 years; pediatric parents: 39.1 years; *P*<.001). Patient demographics with respect to insurance, educational attainment level, and race are presented in Figure 1.

**Table 1:** Respondent demographics.

Demographics		Overall (n=54), n (%)	Adult (n=35), n (%)	Pediatric (n=19), n (%)	*P* value
**Race, n (%)**					.11
	Hispanic	6 (11)	2 (6)	4 (21)	
	Asian	2 (4)	0 (0)	2 (11)	
	Black or African American	33 (61)	23 (66)	10 (53)	
	White	11 (20)	8 (23)	3 (16)	
	Multiple/other	2 (4)	2 (6)	0 (0)	
**Sex, n (%)**					.01
	Female	35 (65)	27 (77)	8 (42)	
	Male	19 (35)	8 (23)	11 (58)	
Age in years, mean (SD)		54.96 (20.48)	63.60 (19.74)	39.05 (9.30)	<.001
**Insurance type, n (%)**					<.001
	Private	20 (37)	10 (29)	10 (53)	
	Medicare	25 (46)	25 (71)	0 (0)	
	Medicaid	8 (15)	0 (0)	8 (42)	
	Self-payer	0 (0)	0 (0)	0 (0)	
	Unlisted	1 (2)	0 (0)	1 (5)	
**Highest education level, n (%)**					.09
	High school/less	16 (30)	14 (40)	2 (11)	
	Some college/associate degree	13 (24)	7 (20)	6 (32)	
	Bachelor’s degree	13 (24)	6 (17)	7 (37)	
	Graduate or professional degree	12 (22)	8 (23)	4 (21)	
**Primary language, n (%)**					.35
	English	53 (98)	35 (100)	18 (95)	
	Spanish	1 (2)	0 (0)	1 (5)	
**Prior telemedicine visit type(s), n (%)**					.03
	Phone	17 (31)	12 (34)	5 (26)	
	Video	22 (41)	10 (29)	12 (63)	
	Phone and video	15 (28)	13 (37)	2 (11)	

### Patient Definition of the Digital Divide

I think the digital divide is all the things that COVID has exacerbated. People with money versus people without it. Having access to the internet, makes you able to do all this stuff, and not having access it's harder to do”

Patient 28

When asked to define the “digital divide,” the majority of patients were initially uncertain of its exact meaning. However, after prompting patients with the definition from our interview script, many recalled personally experiencing the divide and witnessed its effect on family, friends, their communities, and society.

While offering their own definitions of the divide, many patients spoke about the inevitability of technology in every facet of modern life:

Having internet is almost, it's not a right or anything, but it's fundamental in today's society, especially with COVID.

Patient 28

Many felt the growing prevalence and necessity of technology in society. Patients recognized how the differential access and ability to use technology created and continues to widen the chasm between those who can and cannot use technology:

Well, it's not a good thing because we all know technology is coming faster and faster. To be divided and have people who don't know, it's really not a good thing, because if we are going to be using technology for our medical and our health care, we need to know these things.

Patient 50

**Figure 2:**
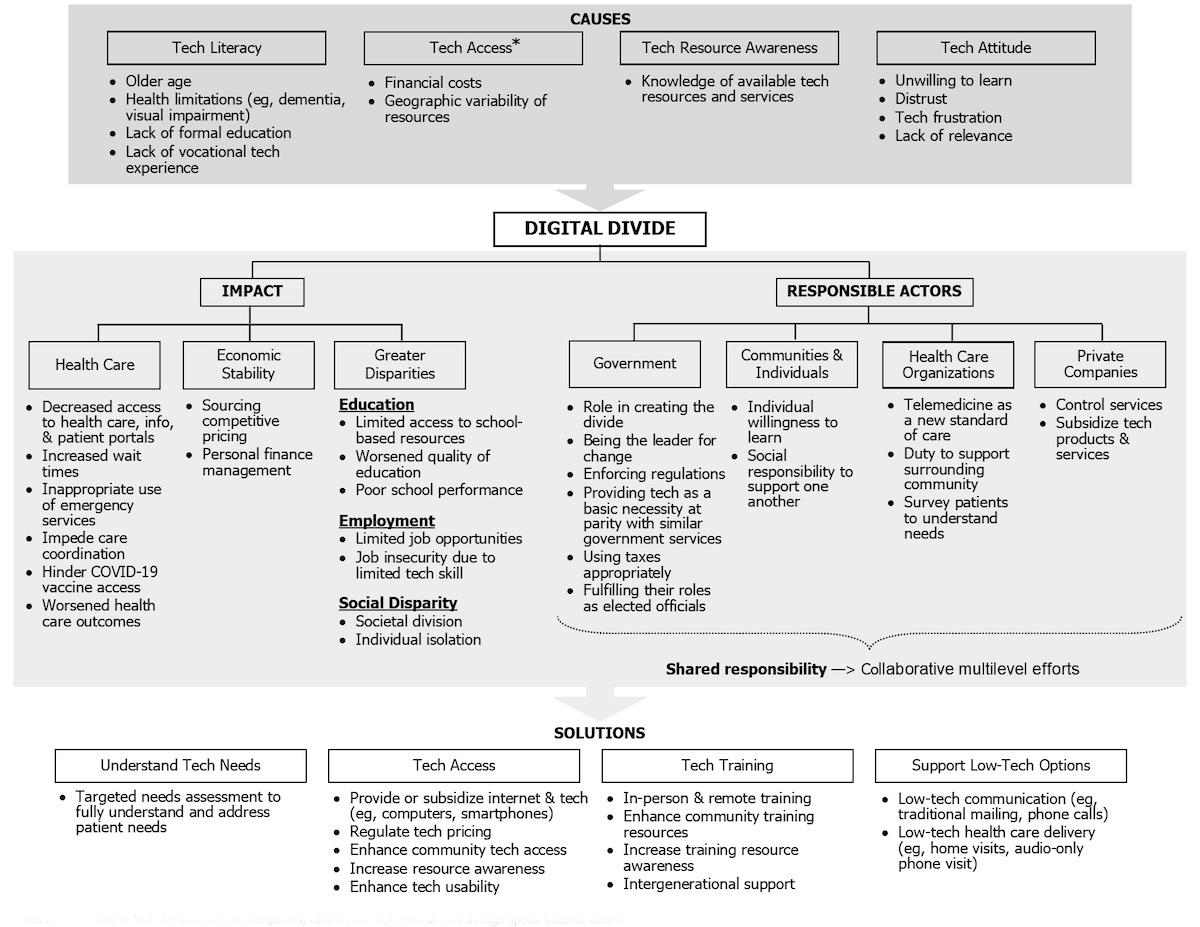
Patient perspectives on the digital divide: causes, impacts, responsible actors, and potential solutions. *Tech access refers to tech devices such as computers, tablets, smartphones as well as high-speed internet access.

Having defined the digital divide, 4 overarching themes were identified in the analysis of interview data relating to its (1) causes, (2) potential impacts, (3) responsibility for addressing, and (4) potential solutions. These are explored in more detail below with their respective subthemes and representative quotes in Multimedia Appendices 2-5 and illustrated in Figure 2.

### Causes of the Digital Divide

Four themes on the causes of the digital divide emerged (Multimedia Appendix 2 and Figure 2): limited technology literacy, limited technology access, unawareness of technology resources, and negative personal attitudes toward technology.

#### Limited Technology Literacy

Beginning with technology literacy, the most common subtheme cited how advanced age is a major contributor and limitation to their ability to learn and navigate technology. Younger and older patients alike recognized the pervasiveness of technology in many aspects of life, not just in health care. They recognized the challenge for older individuals to keep up with an increasingly technology-dependent society in which computers and smartphones evolve so quickly:

At some point, everybody's going to die off who doesn't know how to use them. We got two-year-olds who know how to use it. It's like, eventually, I'm the only anyone who doesn't know how to use it.

Patient 45

Cognitive and medical impairments, including memory loss and hearing and visual impairments, were also challenges that contributed to the digital divide among older individuals. One such respondent with dementia explained how her cognitive illness contributed to her isolation from technology and, in turn, where she stood on the digital divide:

You know I have dementia...I don't know how to do it. I just don't, and it just bothers me sometimes I just can't. It is very frustrating...I can't put it in words right now but it does make you feel different, with all this technology and not enough personal contact. Well, I can't stop it. I wish it was simple, but it's not going to get better. I can't do what I can't do. If you're not caught up, if you don't know how to do it, sometimes you miss a lot. I think it's unfair. Most people don't want to be a burden to the government or their family either way. They just want to take care of themselves as best they can.

Patient 36

Additionally, patients who never had formal training (subtheme 4) or vocational exposure to computers (subtheme 5) found it challenging to stay connected with the rapidly changing nature of technology. These individuals struggled to understand basic technology functions such as sending an SMS text message and were left behind, widening society’s digital divide.

#### Limited Technology Access

The second major cause of the digital divide identified by patients is limited technology access, caused by high financial costs and geographic variation of available services. Patients shared that purchasing technology or an internet plan was a heavy financial burden, particularly for families with multiple children. In other words, cost greatly limits technology access:

I mean look at these kids now that are in school. That's why they need to go back to school because you've got kids or multiple children that are supposed to be on the computer in their relative classroom. They have four, five, six kids. They can't share the same computer. They have to be in class at the same time and these parents, who has enough money to get three or four computers around the house? Really, realistically you don't unless you have some nice money. You don't have money for that. No. My sister's a teacher and she's at home trying to be a teacher and then the kids, they have to go to school. It's crazy. [chuckles] It's crazy.

Patient 6

Patients also discussed the disparities in high-speed internet access between rural and urban populations:

It's socioeconomics for the most part in an urban area...It's being able to afford it and not having enough public resources out there to help bridge that. If you're rural, that's a whole other set of problems that I'm not as familiar with. I know that there are areas that maybe don't have as much cell connectivity, maybe don't have the same internet options. That would exacerbate it. In this area, it's economic. It's being able to afford it.

Patient 31

#### Unaware of Technology Resources

Many patients were unaware of resources such as free or low-cost internet and smartphones, which was identified as a major contributor to the digital divide:

Well, part of it is economics, but I think another part of it is a lot of people don't understand what's available to them. Because they have programs to cover, but a lot of people didn't know how to take advantage of it. They were just out there on their own with nothing.

Patient 17

#### Negative Personal Attitudes Toward Technology

Many patients stated that older individuals are generally reluctant to use technology. Older patients were often frustrated with learning new technology, had feelings of distrust, and were hesitant to integrate it into their lives particularly when the majority of their daily functions did not require technology use. However, even though less technology-savvy older individuals avoided technology use, they did recognize the way of the world was now increasingly technologic:

I think when you didn't get in on that ground floor, some years ago when they started coming and they started coming so fast, it put a little fear into them, and they're like, ‘I don't want to be bothered with that.’ It just took off in a whirlwind and went faster than they were going. I'm guilty too. Probably when that was going on, if I had been saying to my mother, ‘Oh, come on let me show you, let me show you.’ She might be a little more open to it. I have a friend, a very good friend, her dad is 98. He lives on his own, his mind is clear, doesn't have a lot of illnesses. It's like, ‘I don't want to be bothered with that.’ Now they don't have the confidence. It's like, ‘Oh, I'm too old for that.’ That kind of thing. I'm too old for it too.

Patient 50

As a result, patients believed that the digitally disconnected and less knowledgeable individuals were at a disadvantage, more isolated, and left behind.

Society has not accepted the world of non-technological people. I think that they are missing out and I'm really big on what's going on with them, with the seniors. They don't have people to do it for you. My dad has his children, my aunt doesn't. I have done a lot of things for everybody up until the digital thing prevented me, it hold me down.

Patient 19

### Impacts of the Digital Divide

Patients described the digital divide impacting (1) health care, (2) economic stability, (3) education, (4) employment, and (5) social disparity (Multimedia Appendix 3 and Figure 2).

#### Health Care

Eight subthemes were identified related to the impact of the digital divide on health care (Figure 2). Most were related to the expansion of technology-dependent virtual care during the pandemic and how low technology resources and literacy hindered the ability to engage in necessary health care activities such as video visits. One patient stated:

That would mean that there's a lot of individuals who are not receiving adequate medical care right now during the pandemic because they don't have access to those resources.

Patient 10

Some patients felt the digital divide also increases visit wait times. Specifically, they believed that those who are able to conduct virtual visits have quicker access to care including responses to their patient portal messages and increased availability to virtual visits. Conversely, those who are unable to access these online resources had to wait for return phone calls and in-person visits, which are not as readily available.

There were concerns regarding inappropriate overuse of emergency services. Without a virtual option, patients will be more likely to visit the emergency department, which increases the hospital’s burden and possibly inappropriate care:

It would probably cause maybe overpopulated emergency departments because now instead of being able to have video visits. I probably would think that it would lead to people not really getting the health care that they need.

Patient 35

The digital divide can also limit access to online patient portals. Without access to these tools, less technologically able individuals experience challenges in care coordination such as scheduling visits, communicating with their clinicians, and facilitating referrals and tests. Patients also remarked that these portals are not just useful for themselves but also for children’s and elders’ parents and caretakers. Furthermore, lack of internet access impedes general knowledge seeking and access to online, reliable, and high-quality health information.

Patients also found that the divide can worsen personal health care outcomes because of limited opportunities and resources to coordinate health care needs. Lastly, patients recalled how limited low-tech outreach efforts for the COVID-19 vaccine and online scheduling portals posed significant challenges for less technology-savvy individuals:

They (seniors) can't handle that, it's too much. I think that senior people should have a little special consideration seeing that because of the fact that a lot of them are not technically technology-oriented. Why you don't call them, and tell them when they can get an appointment for the vaccine? Why do they have to go online and look for an email, when they don't do computer? See, I have somebody facing this right now.

Patient 19

#### Economic Stability

One subtheme focused on how the internet allows individuals to quickly source and compare prices of basic goods and services. The second subtheme was the impact of the digital divide on the management of personal finances and accounts, which are predominantly done online today. For example, some patients noted that during the pandemic, managing aspects of their basic utilities were only accessible online, which created a frustrating and unfair experience for digitally disconnected individuals and families.

#### Education

Impacts of the digital divide on children’s educational experience were especially prominent due to remote learning during the pandemic. During the pandemic, the lack of access to on-site school–based computers and internet limited children’s ability to gather resources needed for their learning and school work:

We had kids who don't have computers, whose moms were sitting outside the school just so they could have WiFi so their kids could learn.

Patient 45

Society’s increasing dependence on technology also increases financial burden on families with several children. Larger families with multiple children need to spend more to provide basic educational supplies, which are now typically expensive technology items and services. Patients discussed how the divide impacts the quality of education in socially and economically disadvantaged areas, contributing to poor academic achievement. Several patients spoke about how children in communities of color, particularly those on the South Side of Chicago, are more technologically challenged than other children from privileged areas due to less exposure to computers. Thus, the transition to an entirely technology-dependent remote learning environment during the pandemic further exacerbated these disparities and resulted in poorer academic performance:

Well, the (South Side) kids, they have a harder time with just being on the computer. I'm sure some of them have figured it out, but on a day-to-day basis, even in school, they're not on computers like some kids in the suburbs and kids who are more privileged. They're far ahead of some of our kids. This has put our kids behind. That's what I feel.

Patient 47

#### Employment

The inability to access the internet also limits employment opportunities such as limiting one’s search for jobs and professional networking. This reinforces a societal divide where the less educated and technologically able are limited to lower paying, more manual jobs:

It's a privilege to be able to have technology and have that resource readily available. Those who are able to have access to that, it's in a sense like a sense of superiority. It's just a complex of it's a hierarchy in a sense where more people are more educated and those who are less educated. When you're more educated, you're able to be more involved or get higher-paying jobs. In that manner, it creates a divide and a conflict.

Patient 53

Furthermore, the divide threatens job security. Patients noted employers prefer to retain more technology-savvy employees, and computers and machines increasingly replace manual workers.

#### Social Disparity

Lastly, patients highlighted how technology contributes to societal disparity by promoting division and isolation of those that are less technology savvy. More recently, the pandemic and an increasingly technology-reliant society have further isolated and ostracized digitally disconnected individuals:

I think (the digital divide) it's all the things that COVID has exacerbated. People with money are able to get access versus people without it. It's basically having access to the internet, makes you able to do all this stuff, and not having access it's harder to do, I think. I think we're seeing what that means, but I think it does exacerbate conditions like whatever the current state is and just speed things up.

Patient 28

Patients also highlighted the duality of technology: technology can positively and powerfully empower the literate while repressing and leaving the less tech savvy with a more difficult life:

That would set a lot of people apart. It really divides people because you've got to have access to a computer now in order to do the smallest things. You really do. You've got to log onto this to do whatever the application-- I haven't been to the library in a long time, but I bet you have to have a computer now in order to do some things at the library. What's it's going to do I think it's really going to divide people pretty soon if technology can change the world, it's just me saying this, but I think that if technology can change the world the way it is...I think it'll really put a rift between, instead of the high class and the low class, it would be a divide between the illiterate and the literate. Computer literates and illiterates and literates. Well, I think it means that you're dividing to go into two classes. One that is more privileged, probably have an easier life than the other.

Patient 37

### Responsibility for the Digital Divide

Patients had clear perspectives on whose responsibility it is to address the digital divide, and these were organized according to 6 main actors and their explanatory subthemes (Multimedia Appendix 4 and Figure 2).

#### United States Government

By far, the US government was thought to be primarily responsible for addressing the digital divide. Patients believed the government played a role in causing the digital divide, thus they should address it. Additionally, patients told us that only the government has the power to legislate and enforce regulation to protect individuals from unfair business practices:

I think that has to do with the government. I think that's a government issue because I think they the ones that could actually make them (companies)-- they could actually put a cap on all of this.

Patient 2

Several patients emphasized a subtheme related to the need for the federal government to stand out as the leader for change and initiate a top–down plan of action between local governments, businesses, and community organizations to address the divide:

Of course, we know our government needs to be a part of that. That's from The White House, all the way down to our local government. We need those people to be involved because to bring these resources to the forefront, we certainly need money.

Patient 50

With the rising prevalence of technology, patients viewed technology and internet connectivity as a basic life necessity like food, transportation, and clean water. Given that the government addresses issues such as food insecurity, they should be similarly responsible for addressing digital insecurity and providing equitable technology access to citizens:

I guess, yes, if that were a project like the highway system or something, or the interstate system, or health care. It's so central to life. It affects your quality of life if you don't have access to it. It's like the water. It wouldn't be right if people have limited access to food or water. People should have access too.

Patient 28

Patients also believed that elected individuals and taxes should be used to support individuals, communities, and social programs with technology access and training:

We should work to make internet available to everybody. I know the federal tax is designed to do that. We pay extra on our phone bills to pay for internet for people that don't have it...I am definitely of the type that I would rather pay a bit more in taxes and see absolutely everyone have their needs met. I think it's on the government. If we pay taxes, it's for services and being able to function and be in school as you are required to. The government should make that available.

Patient 5

#### Individual Responsibility

Patients recognized that aside from individuals with cognitive impairments, people have a choice in learning how to use technology and seeking access to keep up with the digital world. There needs to be a component of individual willingness to bridge the divide:

It'll still be up to that person if they want to make that change and catch up with the world. It's up to them whether they want to learn. If they don't want to learn, you can't make them. It would be their responsibility. It's just like saying the video visits versus going there in person. If they don't want to learn technology, all right, then you got to go down there in person.

Patient 49

#### Health Care Organizations

Health care organizations were cited as a responsible actor for the divide, especially because technology-based care such as video visits are quickly becoming commonplace and a part of the standard of care. Patients believed that health care organizations have a duty to support their local communities with not only medical services but also technology resources and training because they are in the unique position to directly survey their patients and understand their technology barriers:

Because they're your health care provider. They can't really fix the digital divide that's not related to health care, not the University of Chicago. But in relation to health care, then I think they should be responsible for that. Because I know that a lot of the seniors are not getting the same, because of the fact that they are not technological.

Patient 19

#### Private Companies

Patients expressed that private companies should address the digital divide because they control technology services and resources and have the potential and responsibility to allocate them fairly. Several patients also recalled how private companies denied or did not offer subsidized services to individuals who needed financial assistance. Specifically, one patient stated that part of their own internet bill payments could and should be used to help support services for others who cannot afford internet plans:

Maybe individuals can put pressure on the companies, or we can in our bills agree to pay more because- to help reduce the cost to make sure so that people provide it but then have people sharing, which I think is the next best thing.

Patient 46

#### Communities

Patients expressed the need for social responsibility and technologically able family and friends to support individuals who struggle to access and use computers and smartphones:

I think that the community where we live at. There are programs out here, it's resources that assist with computer classes that get people used to using a computer or even just to know that normal functions of a computer, community resources to assist with that.

Patient 20

#### Shared Responsibility

Lastly, there was a profound recognition that the divide is a real and serious threat to the well-being of our communities and nation. Given the scope and significance of the divide, everyone, including the government, organizations, companies, communities, churches, libraries, and individuals, plays an important role in addressing it:

The digital divide will have to be fixed by everybody. Just like anything else; in the US, when it's time for the big push, everybody has to cooperate. Not just one or two people, or one or two agencies. Government can't do everything. If they could, honey, we'd be a Socialist Party for real and this would be France, but they can't do everything. It's going to take everybody. Every church, every institution, every computer company. The digital divide is not a joke. It is for real just like food deserts; no grocery stores in certain areas for miles, and what if you're on a bus? If you can at least get a phone, then most people can cross that digital divide.

Patient 4

### Potential Solutions to the Digital Divide

Four themes were identified as possible ways to overcome the digital divide (Multimedia Appendix 5 and Figure 2).

#### Understanding Technology Needs

The first and most critical step in solving the divide is understanding it. Patients believed that an initial targeted technology needs assessment of communities and health care organization members would facilitate a baseline understanding of what their unique needs are:

One-on-one surveys with people in various ages and ethnic backgrounds to see how they feel and what their needs are. In order to close the gap, you got to see what you need. You've got to put the information out there and find out what people really need. How many 70-year-olds, or 90, or 40-year-olds need these resources? When you don't know, how can you fix it? You've got to know what a person's needs are before you could fix it. I think starting there, we would get a lot of answers and do things differently.

Patient 50

#### Ensuring Access to Technology

Patients proposed directly providing devices such as computers and smartphones, as well as internet connectivity, to all. Again citing technology access as a basic necessity particularly during the pandemic and for virtual health care, patients called on a variety of actors (eg, government, health care organizations, private organizations, social infrastructure such as schools) to step in and provide the necessary tools to individuals. With many patients unable to afford the required technology to participate in today’s digital world, universally providing the technology is one way to ensure equitable access to everyone:

They have to be able to provide everybody that sort of resource. Just if everybody has equal access to opportunities also that are provided through the internet. Free access to Wi-Fi and providing something to access that, like a computer or a tablet. Providing the resource for these people to be able to access. Even libraries are not enough or some small things like that are difficult.

Patient 53

However, recognizing this may not be feasible. Many respondents proposed providing subsidized technology resources to regulate technology costs, thereby improving access to those who need them most:

Some people can't afford the internet. Some people is just living off of once a month check or some people not getting any income at all, so how would they go about paying their internet bill? To me, for people like that, and people that can't afford it. I think they should have a program for them, where they should be able to get it for free because they know it's a need that they need.

Patient 25

Enhanced shared technology resources in new or existing community settings such as providing free shared use computers or phones in doctors’ offices, public areas, internet cafes, and community centers can help increase technology access:

If there is someplace that we could just use their computers that are already up and specialized...I would go in a heartbeat. You just need help and when you need help, you do whatever you have to do...Give us an option of being able to come into a room or some area where you could come in and use their actual equipment which might be better for all of us.

Patient 6

Many patients specifically commented on how libraries can help bridge the digital divide because they are widespread, play an active role in underserved communities, and offer open access to technology devices and internet connectivity. However, due to COVID-19, patients expressed frustration in decreased library availability and called for the need to re-evaluate and expand library services, such as revising opening hours, providing socially distanced areas to use technology devices, and providing patient care pods where virtual visits could be privately conducted:

Everything has changed. They need to be aware. The library is open from 10:00 to 6:00 or 10:00 to 5:00. Like I said, the library used to be open up to nine o'clock at night, but I know it's not anymore. I need my Zoom (video visit) call to be at 11 o'clock. Why? Because the library doesn't open up until 11 o'clock. I know McDonald's is open, but McDonald's you can't sit in McDonald's now. There's no place that you can go and actually sit but the library. You can only be in the library a maximum of an hour, I think. You know what I'm saying? It's a lot of work but--It's important to keep people alive because I think that's what our main focus should be right by now is to keep people alive, healthy and safe.

Patient 48

To ensure access to technology, there needs to be an enhanced awareness of one’s available resources. Lack of technology resource awareness can be just as prohibitive a barrier as not having the device itself. Again, with closures and accessibility restrictions due to COVID-19, overcoming the digital divide requires active communication of available technology resources to connect them with the individuals who need them most.

And lastly, patients expressed that improving the patient-facing functionality and usability of technology in health care would facilitate technology usage. Suggestions such as more patient-friendly online portals, the ability to share visits more easily, granting portal access to more than just 1 individual (eg, to 2 parents), and video visit platforms that were easier to navigate were all cited as potential solutions to bridging the technology divide. Improved user interfaces could improve how patients use these tools in their own care or to assist family and friends with their care.

#### Technology Training

The second overall solution theme to the digital divide was the provision of technology training to improve digital literacy. One subtheme called for in-person training because it is more relatable and easier to understand. Patients emphasized that the instruction needs to be “simple, simple, simple. It got to be simple (Patient 36)” particularly for older adults or individuals with cognitive impairments such as memory loss. Patients recommended educational institutions such as universities and health care organizations as good venues for hosting workshops, ongoing classes, and even a dedicated technology help desk in clinics where patients and family members could learn how to navigate their online patient portals in-person, conduct a video visit, and use technology in general:

They have classes for everything at the university. Just like they set that up, set a class up. When people come in the hospital, they can go, “Oh you know what? Oh, they having technology class. Since I'm here at the clinic that day, oh let me see they had a class that day?

Patient 32

Additionally, synchronous (eg, a phone line) and asynchronous (eg, preparatory instructional videos and written information) remote learning resources can help patients overcome technology issues related to video visits or the use of patient portals. To troubleshoot potential challenges in advance, some also suggested virtual practice sessions and opportunities to access their video visit platforms prior to their actual appointment. Of all the proposed remote assistance recommendations, the most common was the establishment of a dedicated technology helpline and help desk for patients, particularly because it can be difficult to reach someone to resolve technology issues via the main clinic line:

It’d be helpful if the doctor probably had a tech department...Where you don't have to wait for your doctor to call you back or your nurse to call you back in regard to getting on to your appointment.

Patient 54

Intergenerational help from technology-savvy family members such as children and grandchildren was another commonly cited way to overcome issues with technology literacy. Many patients, particularly older adults, reported having a greater dependency on tech-savvy younger relatives and friends, highlighting the importance of intergenerational assistance to help less technology literate patients navigate technology:

I can't do the latest model phone they have out. My granddaughter won't give me one of those but my great-grandchildren have them. That's who teach me how to work on the computer and it's stuff my great-grandchildren, the little ones. If I have trouble with my video visit, I have two ”greats“ sitting right here.

Patient 8

Similar to the subthemes about enhancing access to technology and increasing awareness of those resources in the community, most patients also emphasized expanding technology training and resource awareness to address digital literacy gaps. Extending this, several patients also envisioned having technology champions and coaches directly in their community. These individuals could then volunteer to use their knowledge of computers and smartphones to educate others in their communities:

It actually has two advantages to it. One, it is creating a job for two people to come in to teach, definitely be the patient that they will benefit from it. It does get another people that will be able to talk and communicate with other people, so they can teach each other because that's how we learn; we learn from one another. If they have a technology class, and maybe they're saying, okay, this is what you need to do to take classes. Maybe they don't know how to set it, maybe I do. Okay, you know what? I'll show you how. The teacher is learning from the students and the students are learning from the teacher too. Being knowledgeable, it balances out and nearly everybody feels needed.

Patient 2

#### Supporting Low-Tech Health Care Modalities

The final theme rested on the recognition that patients who could not or would not cross the digital divide will continue to suffer in terms of their health care access. For these individuals, the need to provide continued equitable access to quality care is paramount. This quality care includes the continued use of low-tech communication modalities such as phone calls and postal mailing to convey important information such as how to get their COVID-19 vaccine.

I think that senior people should have a little special consideration seeing that because of the fact that a lot of them are not technically technology-oriented. Why you don't call them, and tell them when they can get an appointment for the vaccine? Why do they have to go online and look for an email, when they don't do computer? See, I have somebody facing this right now. I think that they should reach out to those people. They shouldn't have to reach out to them. Then some of them are not in line for it because of the lack of technology and I think that's wrong.

Patient 19

Audio-only telephone visits are particularly important for individuals with physical or cognitive limitations, given they described completing a video or in-clinic visit challenging if not impossible.

Some of it is because of COVID. Sometimes it's because the cases it seems, I'm not feeling good and depending on what's going on with us, it can become challenging to get him up and get him dressed and get him to the doctor. It’s (audio-only telephone visits) talking to your doctors, it's not a joy call. You know what I'm saying? This is not where you stand up to go out to dinner, we're talking about life and death here.

Patient 2

Furthermore, low-tech health care delivery in the form of home visits is an important adjunct to care for select patients, and provided a critical health care lifeline that should be supported beyond the pandemic:

My dad and I live together and he's elderly, he struggles with traveling. My thing would be something that would be available for those who are disabled, obviously the elderly who are not in a nursing home or hospice. You know what I’m saying, nursing home care or independent care where professionals can come in and provide the vaccine. They can set up appointments. Essentially, it would be like DoorDash but for vaccines.

Patient 48

For many, the pandemic exacerbated the isolation of older and technology-challenged individuals. One patient commented on the need to not only continue providing these low-tech forms of health care and communication but health care organizations also need to increase their efforts and proactively reach out to our society’s most vulnerable individuals:

This is technology going on, a lot of them are lost. They have no idea because they never worked on the computers. They never had to. Our seniors get lost in the system because they're senior and they're older and they don't have anybody to come and help them. I know somebody that ain't seen their doctor in two years but the doctor never reached out to them either and I was like, well, that's not a good thing. You're the doctor and your patient is a cardiac patient so if you haven't seen your patient in three months wouldn't you have your nurse call and say, ‘You know what we ain't see miss so an so because she has respiratory problems cardiac problems. We need to reach out to her. Because maybe there's something going on. If she doesn't have anybody to come see about it and she can't remember to call the doctor because she's feeling bad, then what's going to happen? They'll find her dead in her apartment and then they'll go, ‘Well, nobody called, nobody checked.’ I know this is a lot of work but it's important to keep people alive because I think that's what our main focus should be right by now is to keep people alive, healthy and safe.

Patient 2

## Discussion

### Principal Findings

This is the first study to directly explore patients’ perspectives on the digital divide and capture them qualitatively in their own words. While an individual’s technologic ability and access were noted as fundamental causes of the digital divide, other factors such as lack of awareness of community resources often disconnected support from vulnerable individuals. Furthermore, while advanced age has been cited as a contributing factor to the divide in prior demographic studies, patients also noted that health limitations such as cognitive decline and memory loss increase technologic isolation [7,20-28]. The resultant lack of familiarity and understanding along with rapid advances of technology leave many older individuals and those with cognitive impairments feeling frustrated, distrustful, and unwilling to learn. The staggering prevalence of dementia in our society has left many home-bound and reliant on family and friends for care [40-43]. Given estimates that the number of individuals living with dementia will rise from 55 million in 2019 to 78 million by 2030 and 139 million by 2050, these are important causative factors to account for as our population’s median age and cognitive illnesses continue to increase [44].

Patients also thought broadly about the divide, recognizing impacts beyond health care (eg, employment, access to nonmedical information, and day-to-day functions critical to individual economic stability such as personal financial management). Education in particular was at the forefront of patient’s minds, primarily because of the advent of remote schooling. However, it is important to know there are many studies that have shown a positive correlation between education and quality and longevity of life [45]. Some patients spoke about the “Homework Gap,” referring to the lack of equitable student access to high-capacity broadband at home [46,47]. While 93% of people in US households with school-age children reported their child engaged in some form of “distance learning,” results from the ongoing US Census Bureau Household Pulse Survey showed that children in high-income households used online resources at higher rates than those in lower-income households [48]. When the pandemic began, 15-16 million K-12 students did not have adequate access to the internet, and by January 2021, up to 12 million students remained under-connected [49]. Even when students did have internet, lack of service provider competition in underserved areas caused unfair pricing and digital redlining, which is the systematic exclusion of low-income neighborhoods from fast broadband service [50,51]. Additionally, an estimated 75% of state and local efforts to bridge digital divide enacted during the pandemic to connect students are due to expire in the next 1-3 years [52]. Based on our patients’ experiences and the direct relationship between educational attainment and wellness, it is critical to ensure temporarily connected but underserved students are not left digitally unsupported again [45].

**Figure 3:**
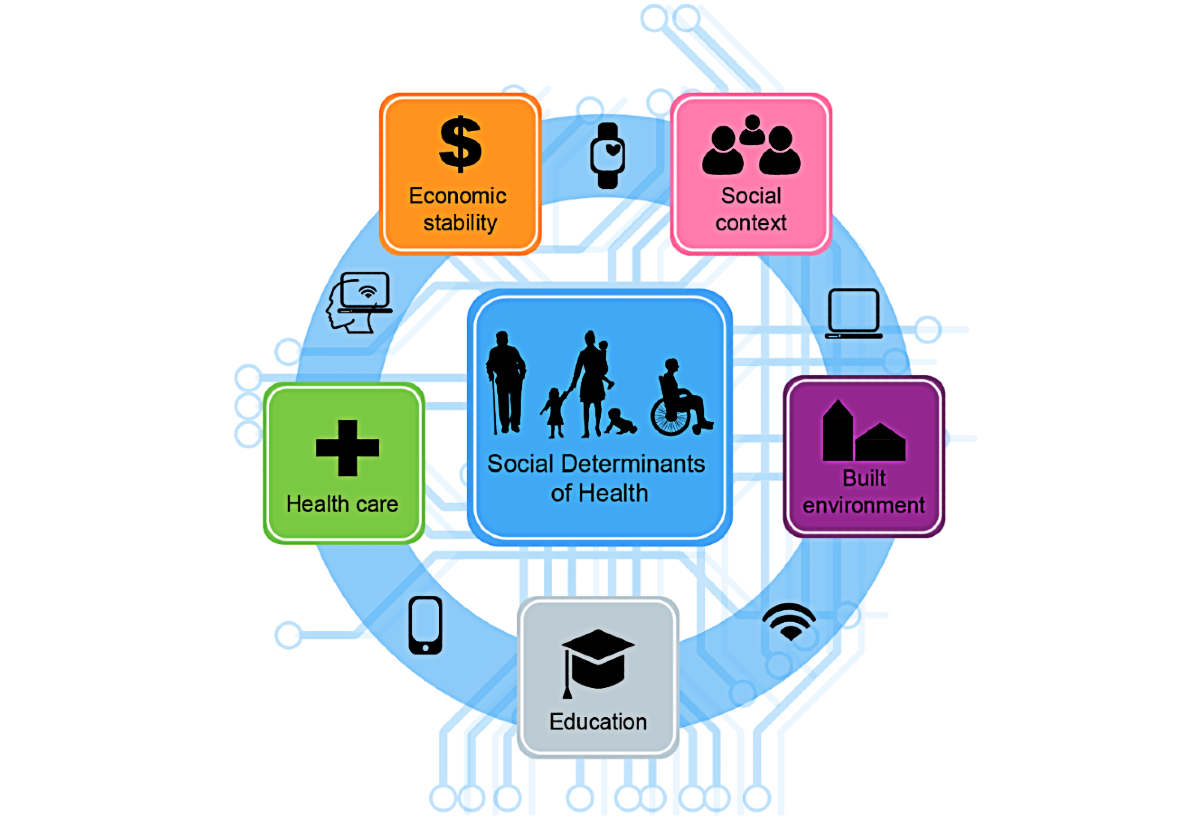
A conceptual model of patient perceptions of technology access and literacy, as it relates to the various social determinants of health.

While technology can alleviate disparities among vulnerable populations, it can also create barriers that perpetuate inequalities [20,53-58]. Patients recognized this dichotomy, and because of our society’s dependence on technology, patients thought the far reaching effects of the digital divide not only worsened pre-existing societal disparities but also excluded and isolated less-resourced communities. As a result, responses illustrated that technology access and literacy were not only viewed as a basic 21st century right, but patient experiences showed it to be also inextricably intertwined with all 5 social determinants of health (SDOH): (1) health care access and quality, (2) neighborhoods and built environment infrastructure, (3) social and community context, (4) economic stability, and (5) education access and quality. Based on our patients’ experiences, it is clear that technology is not the sixth domain on the list of SDOH [59-61]. Rather it is a major controller of every SDOH and the environment in which they can be accessed fully (Figure 3). For example, a stable internet condition and computer are prerequisites for many forms of education. Without these, individuals are unable to take advantage of all that their education offers and often have to undertake cumbersome efforts or greater cost to keep stride with their technology-literate peers. Insufficient technology access and literacy are the drivers of the various SDOH, profoundly influencing whether they are functional or dysfunctional and impacting one’s overall health and quality of life. Examples such as this reflect the modern reality that patients described—one in which technology is a gateway to health and wellness, and thus equivalent to other basic needs—and was the basis for their call for solutions to support technology use.

Patients had insightful solutions to begin supporting technology as a basic need, beginning with obtaining a better understanding of patients’ technical needs. Conducting a needs assessment and screening patients for their technology needs were important in the health care setting, which fits well within the role and function of the medical home [62-64]. Health care organizations are in an ideal position to develop strategies for overcoming barriers to technology in medical care. However, this is not possible until greater knowledge about patients’ technology literacy and access is determined.

A number of creative ways were proposed to support technology access, ranging from direct provision and subsidization of devices to regulated pricing. Patients described the phenomenon of “Parking-Lot Wi-Fi,” which is people sitting in parking lots of shuttered libraries, shops, and schools to connect to their only source of Wi-Fi internet access [65]. Squatting in proximity of public Wi-Fi has become so commonplace that states have begun publishing parking lot maps for residents without home internet access [66,67]. However, even in areas where federal internet service maps indicate broadband access, there are still pockets within these areas that lack affordable, high-speed internet [68]. These communities, known as internet or digital deserts, are often in urban areas, and a Census Bureau Survey showed that 3 times as many households in urban areas remain unconnected as in rural areas [69,70]. Although there are high-cost reforms in places such as the Connect America Fund, which provides funding to service providers that commit to offer voice and broadband services to fixed locations in unserved high-cost areas, digital deserts and redlining continue to exist [71].

Ensuring technology access addresses only 1 facet of the divide, patients recognized improved technology design and training are also critical for improving technical literacy. As an example, patients asked for improved functionality of existing health care technology such as patient portals and video visit platforms. Calls to incorporate the patient perspective in technology design to support not only clinicians but also patients is not a new concept [42]. However, patients are currently asking health care organizations to establish a separate set of resources such as a patient-facing addition of the information technology department to directly support patients with telehealth use. In the face of the digital divide and increasing dependence on telehealth, participatory design and support are more important than ever to consider. Furthermore, given our aging population and the cognitive challenges they often face, providing a simpler and less overwhelming version of these platforms is needed. For example, interfaces with features such as voice activation, memory aids to improve ability to remember important tasks, and easier access sharing with caregivers can make navigation easier and promote a higher level of technical independence [42,72].

Libraries were often cited as a key potential solution to the divide. Libraries hold great promise in helping solve the overall literacy challenges faced by many US adults [73], as well as in addressing technology access and literacy barriers [74-79]. Given their existing social infrastructure and location in communities of need, libraries are well positioned to become part of a more equitable ecosystem of learning, providing access to knowledge, resources, and training that may not otherwise be accessible to people with lower incomes [78,79]. Unfortunately, the pandemic has greatly limited access to many of these spaces and supports, leading patients to ask for a continued re-examination of the role and services that libraries provide. They and other social infrastructures such as churches, senior centers, schools, and parks are important shared spaces in our communities [75]. These spaces have the potential to not only pivot and repurpose existing services but also innovate and collaborate in new ways to bridge the divide and better meet the evolving needs of their communities. To facilitate this transformation, policymakers must invest in efforts such as the E-Rate Program, which was started in 1996 to help public schools and libraries cover the cost of internet access in their buildings. Programs such as the E-Rate can enable libraries and other social infrastructures to take stock of how they are used within marginalized communities and invest in efforts to bring technology access and training to underserved households [76-79].

Beyond providing enhanced technology resources, patients also recognized it was equally important to identify, organize, and connect these services and resources to disconnected individuals. Efforts such as NowPow, a personalized community referral platform that draws on a comprehensively sourced and updated community resource directory, allow clinicians to connect patients to needed medical or social self-care resources [80,81]. Comprehensive, technology-based community asset census mapping will increase awareness of these services and combat the decentralization of community resources by allowing organizations to work synergistically toward their shared goals of individual empowerment and wellness.

Lastly, patients called for the need to support low-tech health care solutions (eg, audio-only phone visits, mailing information, and home visits) in the wake of the digital divide. This was made especially clear by patients who had difficulty navigating video visit platforms and COVID-19 vaccination efforts through patient portals and online scheduling [82]. While some individuals were open to attempting these new and unfamiliar forms of health care, especially if intergenerational support was available, many were not willing or able to. In these situations, it was necessary to support their continued access to care as a basic right for health care equity.

Supporting low-tech, audio-only virtual care has become an increasingly relevant concern, especially if reimbursement parity between telephone and video visits is discontinued and audio-only phone visits are no longer reimbursed [7,23,30,31,83-86]. Furthermore, if audio-only visits are not supported, those unable to navigate a video visit may defer care altogether—a common occurrence among patients during the pandemic [87,88]. Telephone visits support high-quality care, particularly in primary care and community health settings. Since 2010, the Veterans Health Administration has incorporated scheduled telephone visits into their patient-centered medical home model to improve care access and efficiency [84,89]. In studies with seniors and in mental health settings, audio-only phone visits were as effective as video in resolving urgent and nonemergent needs [90,91]. And in safety-net populations, telephone visits during the pandemic increased access to care, reduced wait times, and in certain circumstances, offered high quality of care comparable to that of video visits [28]. While additional study is needed to compare experiences and outcomes of video and audio-only visits, the potential benefits of video visits will not be realized if patients cannot navigate the technology. As such, continued support of low-tech but high-value care and communication is needed. Further, innovative approaches are necessary to overcome some of the challenges of conducting research with socially disadvantaged and technologically isolated groups, to increase their voice and representation in health and medical research [92].

### Limitations

Our study has several limitations which are important to note. While we conducted our study with both adult and pediatric parents, this is a single-institution study and we only included patients that had prior virtual visit experience (phone or video visits), both of which may limit generalizability to other patient populations. It is important to note, however, that surveying this population gave us access to patients who are on both extremes of the digital divide: those that may not have had the technology access and literacy to conduct video visits and had audio-only phone visits, as well as those who were more digitally literate and connected and able to have video visits. Another limitation to generalizability is the large percentage of our study population with higher education, therefore representing a more socioeconomically wealthy group. However, it is important to note that nearly one-third of our respondent population had an educational attainment level of high school or less, and that the majority of our respondents had Medicaid or Medicare insurance. Furthermore, we only solicited the views of primary care clinic patients; results may differ when interviewing specialty clinic patients. Additionally, our low response rate may have contributed to a nonresponse bias. Social desirability and recall bias may have impacted patient responses to our interview questions.

### Conclusions and Future Directions

Patients are keenly aware of the digital divide and how it disparately impacts health, work, education, community and social contexts, and personal economic stability. As such, digital access and literacy are not merely another SDOH, they are in fact drivers of each SDOH and are fundamental to their function or dysfunction (Figure 3). Given the complexity of the divide, a shared responsibility between the government, private sector, social infrastructure such as health care organizations, community organizations, public services, and individual citizens is needed. Community and organizational needs assessments are essential to identify and target the most impactful interventions. Providing technology is a necessary first step; however, solutions must reach beyond access alone. Development and dissemination of technology literacy training programs and increasing awareness and coordination of available resources will take time. Importantly, even with all of these efforts, some will never cross the divide. For these individuals, it is necessary to continue to support low-tech means of communication and health care delivery to prevent further isolation and widening of the digital divide.

Future work is needed to understand the technology needs of digitally disconnected and excluded individuals and communities. As telehealth continues to be integrated into clinical care delivery models, studies should explore how video versus audio-only phone visits impact patient experiences and outcomes, as well as the role of digital navigation to proactively identify and address technology needs. Advocacy efforts should focus on the utilization of public and private resources to address issues such as expanding device and broadband internet access, improving general and health technology literacy, and advocating for policy to continue supporting both high- and low-tech forms of health care delivery for those unable to cross the divide.

## Appendix

Multimedia Appendix 1 Digital divide interview script.

Multimedia Appendix 2 Causes of the digital divide.

Multimedia Appendix 3 Impacts of the digital divide.

Multimedia Appendix 4 Responsibility for the digital divide.

Multimedia Appendix 5 Potential solutions to the digital divide.

## Abbreviations

CMS: Centers for Medicare and Medicaid Services
SDOH: social determinants of health
UCMC: University of Chicago Medical Center
