# Supplementary material for: Digitally Disconnected: Qualitative Study of Patient Perspectives on the Digital Divide and Potential Solutions
Source: JMIR Hum Factors. 2021 Dec 15;8(4):e33364. doi: 10.2196/33364 (PMC8675564; doi:10.2196/33364)
Supplement: Multimedia Appendix 1 [file humanfactors_v8i4e33364_app1.docx]

Multimedia Appendix 1 - Digital Divide Interview Script

| **Digital Divide Interview Script** | |
| --- | --- |
| **Demographics** | |
| 1. **Are you of** **Hispanic, Latino, or Spanish** origin? **a.** Yes **b.** No | |
| 1. **How would you best describe yourself?** | |
| - 1. Black or African American   2. White   3. Asian | - 1. Native American   2. Other: ______________________ |
| 1. **What is the highest degree or level of education you have completed?** | |
| - 1. Less than high school   2. High school graduate or GED   3. Some college but no degree  1. **What insurance do you / does your child have?** | **d**. Associate's degree  **e**. Bachelor's degree  **f.** Graduate or professional degree |

| **Digital Divide** |
| --- |
| **My last set of questions are about something called the digital divide.**   1. Have you heard of the term the **digital divide** before?    1. If YES, **what** does that term mean to you?    2. If NO, say “No problem. Let me explain it for you, ***the digital divide means…”***    3. If they define the term correctly or incorrectly, still explain it to them to set a common language. If they were correct, say something like “Yes, you’re right! ***The digital divide means…”*** If incorrect, “Great, thanks for that. For the purposes of these questions, when I say “digital divide” I’m talking about that ***“…there are people…”***       1. ***The digital divide means*** there are people that have and can use technology like computers and the internet. But there are also people that do not have or cannot use this kind of technology. So there is a split or a divide, between people that have and know how to use technology, and those that do not. 2. Have you or a loved one **personally** experienced this?    - 1. If YES, tell me more? What happened? What did that result in?      2. **What did it affect? Possible prompts:**         1. Your/their **medical care or access** to health care?         2. Your/their **work** **or access** to work?         3. Your/their **education or access** to education?         4. Anything else? 3. What does having a **digital divide in our community or even in our country mean**, if there is a group of people that have and can use technology, and a group that cannot?    1. Tell me more, can you give me examples? E.g., Possible prompts - such as differences in opportunities for healthcare, communicating with family, etc. 4. **Why** do you think there is a digital divide?    1. Tell me more, can you give me examples? 5. Whose **responsibility** do you think it is to deal with or fix the digital divide? E.g., Government, local institutions like University of Chicago Medical Center (UCMC).    1. **Why** do you think it’s their responsibility? 6. **How** do you think **we** can close the digital divide?    1. Can you give me some examples or ideas? Are there ways UCMC can help? Others? |
