# Supplementary material for: Digitally Disconnected: Qualitative Study of Patient Perspectives on the Digital Divide and Potential Solutions
Source: JMIR Hum Factors. 2021 Dec 15;8(4):e33364. doi: 10.2196/33364 (PMC8675564; doi:10.2196/33364)
Supplement: Multimedia Appendix 2 [file humanfactors_v8i4e33364_app2.docx]

**Multimedia Appendix 2 - Causes of the Digital Divide**

| **Theme** | **Sub-theme** | **Affected Group(s)** | **Example** |
| --- | --- | --- | --- |
| **Technology literacy** | Older Age | Senior citizens | That's a part of the experience. Not as hard for kids but hard for elderly. Yes. Not the whole entire community. Just the elderly, the older people. Just because of the age. The technology and the different generations. (Patient 54) |
|  | Health limitations (e.g., dementia, visual or hearing impairment) | Senior citizens; Health impaired individuals | That will definitely be a big factor because like I said a lot of things I forget, you know, I just forget. I guess it's the nature of my illness because I'll be 80 in a few months and I forget. (Patient 32)  Then of course in my dad's situation, he's blind. I have to listen in on certain things, and then he has to call me and then explain to me what's going on and then he tells what website to go to. It's still out of his frame of reference or his frame of understanding that pretty much I can access anything via my phone. (Patient 48) |
|  | Lack of formal education | Technology limited individuals | Because it's not something they grew up with. Even my generation, I'm older than my children, it was just starting with my generation. The young people are completely 100%, that's all they've ever dealt with. When I was I want to say a sophomore in high school, we had to take a typing class on a computer and that was unusual. That's what it is. It's more new to us than it is-- They all know how to do it. We just don't. (Patient 23)  That is why this gap opens because there are people who do not inform themselves, not just that it is poor, but because they simply do not have the education to be able to ... I am missing the word in Spanish...to update in this area of ​​technology, did I explain myself well? We live in a time when I believe that all people should be informed and educated according and know how to use technology, social media and all this. I believe that until there is education in all economic strata, then that gap we are talking about can be broken. (Patient 34) |
|  | Lack of vocational experience with technology | Unskilled workers; Senior citizens | I think it's based on educational divide and economic divide and probably just vocational divide. There are certain types of jobs that you have to use technology for and they often provide training. It's built into your day-to-day. For example, in my job I have to use technology and so I get trained and it's just a part of the job perks. I don't think that's the case for someone who works in the fast-food industry, necessarily. Even there though, and even driving an Uber, you have to have a certain understanding of technology obviously, but I don't think it's equal. (Patient 37)  I don't know anything. I'm not really up on the latest technologies out because it's six to seven years since I retired so I haven't been doing a lot of stuff on a computer or anything at all really. (Patient 32) |
| **Technology access^a^** | Financial costs | Low SES^b^ individuals | Honestly, I think it's related to the community. For example, in my personal experience a lot of the underserved population, that type of community, they just don't have the means or the access to computers. Asking them to make sure they have an internet speed that is fast enough and they can't even keep their lights on it's disproportionate. (Patient 35)  Oh, I know it's a problem in the black community because this generation, I’m gonna say this generation of young parents. This generation of young parents will go out and buy $150 pair of shoes before they would go out and buy their children a laptop or a tablet to be able to have the technologies that they need to have. I think if the younger generation would get more involved in buying the children or buy themselves the tools that they need to work with to be able to keep up with this technology, this space that we're living in, they would be much better. (Patient 2) |
|  | Geographic differences in resources | Resource poor rural areas; Urban internet deserts | I think part of it is just development. Like I said, rural areas are also left behind with the whole digital divide. It's not just poor people. (Patient 28)  In some neighborhoods, there are those "deserts", but how do you deal with that? (Patient 4) |
| **Technology resource awareness** |  | All | Something we went back to in our earlier conversation about having the availability to acquire those kinds of devices and also the training. A lot of people, they don't have the equipment, and then they don't have anybody to show them or to help them do it or even know where to go to get it. To be divided and have people who don't know, it's really not a good thing, because if we are going to be using technology for our medical and our healthcare, we need to know these things. I think in all our communities, we need to reach out to those people and make these resources available to them. Like, "Hey, you could get a phone or you could get a computer or we can teach you how to use these things. Here's a number you can call if you need help, we can walk you through it." (Patient 50) |
| **Technology attitude** | Unwilling to learn | Senior citizens; Technology limited individuals | Well, I kind of already see that because, with the older folks in the community, I've dealt with, like my parents and so on, they don't deal with computers. I remember back then just even having a working VCR for them and they don't want to learn. They've already dealt with all that, the old school labor and cleaning and so on, they don't. My mother refuses to learn. I think we finally get her a cell phone and she's in her 70s. When I say, "Mom, text me the picture of a question she had." "I don't know how." "Will come to have teach you." "I don't want to." It is an issue and I feel bad because they don't want to learn. They've already done then, it's harder for them. It frustrates her, I could tell. (Patient 51) |
|  | Distrust | Senior citizens; Technology fearful individuals | Because some of the older people feel that all this stuff is all messing things up. A lot of people are anti-internet. They don't want to because of security. A lot of hacking events, they scared of that. They don't want to be bothered with that, and that's somewhat age-related. (Patient 19) |
|  | Frustration with technology | Senior citizens; Technology limited individuals | You talk about terrified. I was terrified to try and use a computer because, first of all, I don't want to remember any more numbers. I don't want to remember changing the password every 30 days. I hate-- That's one of the main reasons that I hate computers. Let me change my password if I want to, to a simple password. Don't tell me that I can't add a number on the end, like I said a number on the end [laughs] my password is like place 1. Let me say place 2. They'll tell you, "No." It's just like, "Oh my God, from now I've got to try and remember whatever, something totally new, random.” (Patient 6) |
|  | Lack of relevance | Senior citizens; Technology limited individuals | If somebody is going about their business living their life and they don't have technology, they don't need the technology. What do they need the technology for? If they've gotten this far without using it and they don't want to use it, then what's to be? (Patient 45) |

^a^ Includes computers or tablets, smartphones and internet access

^b^ Socio-economic status
