# Supplementary material for: Digitally Disconnected: Qualitative Study of Patient Perspectives on the Digital Divide and Potential Solutions
Source: JMIR Hum Factors. 2021 Dec 15;8(4):e33364. doi: 10.2196/33364 (PMC8675564; doi:10.2196/33364)
Supplement: Multimedia Appendix 3 [file humanfactors_v8i4e33364_app3.docx]

**Multimedia Appendix 3: Impacts of the Digital Divide**

| **Theme** | **Sub-themes** | **Affected Group(s)** | **Examples** |
| --- | --- | --- | --- |
| **Healthcare** | Inability to access care | Senior citizens; Technology limited individuals | “Yes, it most definitely do (impact healthcare), and the fact that they don't really go to the doctor like that (by video visit) anyway. It's most definitely a problem, so yes, it is. They would be highly impacted because like I said, they are a part of that digitally divided where they not really into technology. If they have to have a video visit with their doctor, but they can't because they don't have internet, then how would they go about doing it?” (Patient 49) |
|  | Increased appointment wait times | Senior citizens; Technology limited individuals | “It does (impact healthcare). She's an oncology patient so if she has technical difficulties with her virtual visits, she has to wait until a new slot opens up for her to go in person or wait for a phone call follow-up.“ (Patient 10)  “I was told I couldn't get an appointment with my doctor for two weeks but that I could have a video call with her that same day or the next day. Sometimes it can be difficult to get in to see the doctor.” (Patient 37) |
|  | Inappropriate use of emergency services | Individuals; Healthcare Organizations | “Maybe overpopulated ER because when people by the time that they're so sick, they probably go to the ER because they haven't been able to see a doctor that they need to see.“ (Patient 35) |
|  | Access to online patient portals | Senior citizens; Technology limited individuals | “Yes, it does. Having the issue-- my father's trying to get in touch and he's 85. My aunt-- they don't do computer like that. We're not even logged into MyChart. We don't even have a MyChart access. Actually, I just got one the other day. A lot of that stuff is done online.“ (Patient 19) |
|  | Access to overall healthcare information | Senior citizens; Technology limited individuals | “That would inhibit some people's ability to quality healthcare information. Everything is, "Go online. Go online." Everything is. Some of those people are old-schoolers, they don't know nothing about going online. They're limited in the information they can get. They're limited. They can’t use it.“ (Patient 19) |
|  | Impede care coordination | Senior citizens; Technology limited individuals | “They depend on doctors sending out links about creating new referrals, going to referrals, checkups, setting appointments, canceling appointments, things like that. Granted, they can do it on the phone but I can imagine that it's way easier for them to do so. I just don't think that they understand the possibility that it will be easier for them because they've never had to. They can do it just going into the office or calling the doctor's office but again, they need to call, say, on a Saturday or a Sunday. Like the only person that's available is like a chat service. They're not going to be able to get help unless they have to go to the ER urgent care. That doesn't help. Whereas if you want to send a quick email to your doctor and they might be able to have off days where they can respond to patient inquiries and whatnot, they probably can't do such a thing.“ (Patient 48)  “That's where I've seen their biggest complication. They had a parent who was in the hospital, so they had to rely on their landline at home to be able to be in contact with the parent, to make sure they're okay. Once they left the home, they were now not in communication, because they didn't have a cell hone, where, should their parent need them, could reach them, things like that.“ (Patient 31) |
|  | Hinder COVID-19 vaccine access | Senior citizens; Technology limited individuals | “Somebody in my family and a lot of that appointment, getting that vaccine, you have to be online. Meaning your doctor's not going to call you, you've got to go to MyChart. Some of these people are old, they don't do that, so they can't get the information like you should be able to.“ (Patient 19) |
|  | Worsened healthcare outcomes | Senior citizens; Technology limited individuals | “It would be bad for their health I’m sure because they wouldn’t be able to follow what’s going on with their medical progress as well as other people who have that access could worsen, could potentially worsen their health.“ (Patient 52) |
| **Economic Stability** | Source competitive pricing | Senior citizens; Technology limited individuals | “It's a lot of things. There's conveniences in being online and as a result, when you purchase things online, they're often less expensive than if you were to call or go in-person. Even purchasing a plane ticket online is cheaper than if you have to go to a travel agency or go to, I don't know, an airport to get one. I don't know where you would go if you didn't buy it online. Even having a call. They tell you that it's cheaper online. So not being able to do that.“ (Patient 46) |
|  | Personal finance management | Senior citizens; Technology limited individuals | “Yes, it's terrible because we could go without having-- Really, we didn't have anything, being able to access certain things because you don't have a computer. It was terrible because it was something so simple and I swear it was like a utility company. It was something. I just could not believe it. I said, "Are you serious? You mean to tell me if I can't log onto this, then I'll have no access?" I cannot remember what it was, but I was so disgusted because it hurt me. I'm just like, "Wait a minute. What am I supposed to do?" Just wait for some handouts, for someone to call me and say, "Hey, are you having problems doing this and that?"“ (Patient 6) |
| **Education** | Limited access to needed school-based resources^a^ | Low SES individuals; School-aged children | “It's mostly needing access to the library and school kids needing access to some of the things that school provide like internet connectivity, being able to be on their Chromebook for their school work, maybe even it's a hot meal. In terms of digital divide around here, I live in Woodlawn, so there's definitely folks who are very much relying on the schools to be able to use Chromebooks or internet to keep their kids in class. If you have a slow connection, that kid's going behind minute one that that connection isn't on.“ (Patient 31) |
|  | Lack of financial means to purchase technology devices or internet for virtual learning | Low SES individuals; School-aged children | “I mean look at these kids now that are in school. That's why they need to go back to school because you've got kids or multiple children that are supposed to be on the computer in their relative classroom; they have fourth, fifth, and sixth. They can't share the same computer. They have to be in class at the same time and these parents, who has enough money to get three or four computers around the house? Really, realistically you don't unless you have some nice money. You don't have money for that. No. It's crazy. It's crazy.“ (Patient 6) |
|  | Worsened quality of education | Low SES individuals; School-aged children | “I say, this whole COVID has done something. These kids are dumb. They are so far behind. All these kids need to repeat the grades that they missed, that they haven't been in school for. They need to, but who's going to punish kids and do that? Again, because these kids in these socio-economic hard-hit areas, they don't have access to a computer because they are being stolen, or they are being manipulated in some other way in a bad way. Maybe they're just running numbers from them or whatever. Yes, I mean, what are they going to do? These kids don't have a computer. They're behind. They're really behind, and all the teachers can do is just pass them along, and that's it.“ (Patient 6) |
|  | Poor school performance | Low SES individuals; School-aged children | “You just see the difference in the kids that have this computers or whatever in their home and ones that don't. It's a lot of difference in how they interact and everything.“ (Patient 32) |
| **Employment** | Limited job opportunities | Technology limited individuals | “It limits their opportunities, I think, if you're not online, where you can quickly react if someone emails you an offer, for example. If you're only going to the library once a day or two, you might miss out on an opportunity that was only briefly available. You're also not, I think, as connected in terms of the grapevine. Maybe you're not on the social sites where jobs are being posted regularly and have a regular presence where either you might be found or you might find something. If you're going to the library, you're probably prioritizing what you absolutely have to get done and maybe not hanging out on LinkedIn or something. I think it would limit opportunity.“ (Patient 31) |
|  | Job insecurity | Technology limited individuals | “Another way it would be a disadvantage because a lot of people will lose a job. They do have a lot of people that are computer savvy so they go in and they have some good people. Because most of the time everybody is trying to get cut back on their job, they got robots. They got the computers. All of that.“ (Patient 24) |
| **Social Disparity** | Societal division and individual isolation | Technology limited individuals | “That they're really ostracized and that they're separated from most of the society, because most of the things we do, things are digital.“ (Patient 17)  “It just wouldn't be fair. If only half the people knew how to use the computers and all this stuff, and the other half didn't, then it would be a slow down, because the ones that know how to use the computer, it's not going to just take the time to show the ones that don't know how to use a computer. They don't want to take the time to teach them. You got a have and a have not. It's just not fair.“ (Patient 44) |

^a^ Includes school-based computers, tablets, and internet access
