# Supplementary material for: Digitally Disconnected: Qualitative Study of Patient Perspectives on the Digital Divide and Potential Solutions
Source: JMIR Hum Factors. 2021 Dec 15;8(4):e33364. doi: 10.2196/33364 (PMC8675564; doi:10.2196/33364)
Supplement: Multimedia Appendix 4 [file humanfactors_v8i4e33364_app4.docx]

**Multimedia Appendix 4: Responsibility for the Digital Divide**

| **Actor** | **Reason** | **Examples** |
| --- | --- | --- |
| **United States Government** | Role in creating the divide | “I think it's the federal government, probably, and the local government. I think they both share the blame and the responsibility to make these things available to people who don't have the financial means.“ (Patient 48) |
|  | Enforce regulations | “They (government) do have the power. They do have the resources to be able to put-- We put all of these people in office to make the law and whatnot. Because we put up an office and whatnot. They know that everybody doesn't' have the same kind of money. You know what I'm saying?“ (Patient 2) |
|  | Being the leader for change | “I think it needs to come from the federal government on down. I think it's on the government. The government should make that available. Same with healthcare. I honestly think healthcare should be just a right, a human right. When it comes down to civil rights and human rights, we look to the federal level for that level of protection.” (Patient 31) |
|  | Provide technology as a basic necessity at parity with similar government services | “Well, really it makes sense, we as senior citizens and we on limited income. I feel like if this is really necessary and the medical fields seems that we can have this, I think the government should step in and take over and do something. Because they should be able to provide equal access to education and since education is online right now, they have to be able to provide everybody that that sort of resource. Just if everybody has equal access to opportunities also that are provided through the internet.“ (Patient 13)  “I guess because it's that thing that's like a welfare issue, and it's something basic and relatively inexpensive, that could really improve the quality of life for a lot of people.“ (Patient 28) |
|  | Use taxes appropriately | “I think it should come from the federal level. I don't think we should be waiting for rich institutions and people to give us handouts. We pay taxes. Taxes should cover these things.“ (Patient 31) |
|  | Fulfill the roles of elected officials | “I think the politician (is responsible). When you throw people up in those positions to fight for you and they don't, they're only in it for the money, I put a lot of blame on them because we voted for you because you promised to do these things. Once you're in the office, it's like, ‘Well, I'm here now.’ That's it.“ (Patient 44) |
| **Individuals themselves** | Willingness to learn | “I believe that the people who can access well, but those who cannot, I believe that we have no excuse for having that gap, because we are again in a country where there is all the aid. I think that people who say, ‘I can't, video chat is complicated for me," I think it's an excuse simply not to modernize or to move forward.’ (Patient 34) |
| **Healthcare Organizations** | Telemedicine as a new standard of care | “If the video visits and telephone visits or whatever are going to be a new standard of care, then I think it would be the provider's job or hospital's job or whoever's job to make sure that if they are going to offer these patients video visits for that or telehealth visits, that they have the appropriate resources to access them.“ (Patient 35) |
|  | Duty to support surrounding communities | “(Health care organizations) could do sliding scale, perhaps, for families that maybe can't afford. Maybe make sure that people in their area can have access, and do it at a sliding scale or something along those lines. An institution and its donors could certainly come up with a campaign to raise funds to make sure that everyone in their surrounding communities has access to healthcare at the neighborhood hospital.“ (Patient 31) |
|  | Survey patients to understand needs | “They (a hospital) could think, ‘We're doing this and we're doing that.’ Maybe you're just doing what somebody in the office thinks needs to be done and really not looking at the bigger picture because everybody ages every year. Next year, somebody's going to turn 80, and 90, and 70, and 60, so you've got to- just something out there to say, ‘Oh, you got a hundred 60-year-olds that really don't know how to use the internet or they're struggling with that." It's going to have to be finding out what's needed out there and who needs it.’ (Patient 50) |
| **Private Companies** | Control services | “It’s the money and certain services. The reality is that they're companies that own the resources and they're able to choose what they want to pay and what they require for access, which is unfortunate and some people can't afford that access.“ (Patient 46) |
|  | Responsibility to provide services to those that can’t afford it | “Because they have it. It'd be like if I own Lake Michigan and that's the body of water that feeds the Chicago water source, and then I only allow those who could afford to pay me my rate to have water but that didn't represent every single body in Chicago, then that means that there would be people in Chicago without access to my water. It's not like they can go anywhere else, so then they'd have to rely on the kindness of those who can afford the water to share with them. I would think that I would bear more responsibility to make sure if I have this resource and I'm the ultimate control of it, then I should be making sure that everybody has access to it.“ (Patient 46) |
| **Community** | Social responsibility to help one another | “It's a social responsibility. There's numerous factors. If those individuals are receiving any sort of government assistance, that's something that can be offered, a program that can be offered through that, because we know they're on a fixed income, and then also family members. Then I'm quite sure a lot of individuals in the community have home health care aides and the free wifi or the cell phone that can be provided to individuals, not just for the senior community, but there are also other families who are unable to have that access.“ (Patient 10) |
| **Shared responsibility** | Collaborative multi-level efforts | “It takes a village, as they say, to raise a child. That’s the same thing. It’s a village with everything else, with the process. Of course, it starts with the President, then the government, then institutions, then everybody else can play a part in it.“ (Patient 7)  “I think local government and the internet providers should have to work together on it. It's a service in the community. I think that having those two providers come together to serve the community that they work in is essential.“ (Patient 26) |
