# Supplementary material for: Digitally Disconnected: Qualitative Study of Patient Perspectives on the Digital Divide and Potential Solutions
Source: JMIR Hum Factors. 2021 Dec 15;8(4):e33364. doi: 10.2196/33364 (PMC8675564; doi:10.2196/33364)
Supplement: Multimedia Appendix 5 [file humanfactors_v8i4e33364_app5.docx]

**Multimedia Appendix 5: Potential solutions to the digital divide**

| **Theme** | **Solution** | **Actor** | **Example** |
| --- | --- | --- | --- |
| Understand technology^a^ needs | Targeted needs assessment | Government; Healthcare organizations; Community organizations | "Those who said they are having difficulties accessing online visits, ask them more. Is it because you don't have a device? Is it because you don't have Wi-Fi? We have this option, you can come and visit us or we could provide you with a tablet. The questions you asked me are questions that should be personal to the people that are being treated. In that way, they're able to adjust to personal needs. (Patient 53) |
| Technology^a^ access | Provide technology^a^ | Government; Healthcare organizations; Businesses; Social infrastructure (e.g., schools, libraries) | Work with Medicaid/Medicare to do some sort of program to help with seniors and the low-income and the disabled to either to file for being able to get refurbished phones and laptops and whatever else so that people can get access to care. I think that's been the biggest issue...they don’t have access to a lot of things. (Patient 48)  If your child had a report and they needed the internet, if we can have some internet cafés that are public- literally, for the public. People can come in just plot down and either have these video visits, if they're private, like telephone-booth spaces, or be able to work on- search the internet for a job or a research paper or whatever. (Patient 46) |
|  | Subsidize technology^a^ | Government; Healthcare Organizations; Community Organizations | I don't know what the hospital could do anything about that but if they can try to help. If a person tells them, "Well, I don't have anything to do my home visits" or things like that. They should try to see if it's a program or something that could help them pay for it for a month or something like that. I know everybody not going to be able to get the program, but they should look to the people that they truly think that need really actually really help fund this program. And some people can't afford the internet. Some people can't afford it so how would they be able to get it if they can't afford it? Some people is just living off of once a month check or some people not getting any income at all, so how would they go about paying their internet bill? To me, for people like that, and people that can't afford it I think they should have a program for them, where they should be able to get it for free because they know it's a need that they need. (Patient 25) |
|  | Regulate technology^a^ pricing | Government | "Put a cap on how much a phone costs, and how much a tablet costs, and how much the internet service costs. If they would put a cap on all of that stuff, then it wouldn't be so competitive. It's so competitive because (companies) just charge (customers). When you do the research, none of the internet services are giving you anything. It ain't cheap at all. None of it. Everybody does not have the same finances going on. You need to put a cap on it, so even poor people will be able to have the internet and stuff, the sources that they need to work with, as well as rich people. Let it flow all the way across the board, let it be easy so that people will be able to get it." (Patient 2) |
|  | Enhance community technology^a^ access | Government; Healthcare organizations; Community organizations | Provide areas and doctor's offices or even reaching out to public libraries to help provide a space for patient care, like a patient care pod in a public library or something where they can get these services. (Patient 26)  "The library is open from 10:00am to 5:00pm. The library used to be open [until] 9:00pm, but I know it's not anymore... I need my Zoom call to be at 11 o'clock. Why? Because the library doesn't open until 11 o'clock. It only takes a minute to get stressed out." (Patient 2)  I think some of these things that Brown and Black people can't afford ought to be maybe at libraries for people who don’t have the financial means. The city and the state ought to be active in trying to make these things available to Brown and Black people. (Patient 48) |
|  | Increase technology^a^ resource awareness | Healthcare organizations; Community organizations | Because everybody just doesn't have that availability or the finances to do those kind of things. Even not just in this particular case, and not just seniors, but younger people too. Everybody doesn't have the money to go get an iPhone or whatever. It's always good to know a place where you can get some assistance. We all need a little assistance nowadays. (Patient 50)  When an elderly person is scheduled to have a video visit, maybe someone calling them or sending them out information in their MyChart, like, "Hey, here are a list of places that you can go for your video visit to use a computer or to have free Wi-Fi, or this is a place that you can go to get low-cost internet." A simple list of resources for them would help. I think patients probably will be very grateful for that. (Patient 35)  When they do that, they need to put (a list of resources) together, and when they mail that they need to have the addresses, the phone numbers, and also the time because everything has changed now. (Patient 2) |
|  | Enhance technology usability | Healthcare organizations; Private organizations | The three of us on MyChart but my son's MyChart is linked to my husband's MyChart. He's able to access it...When we had to have an appointment before I asked the clerk in front if they can link it on MyChart and then they said they can't. It's only one person and they said just let it be...they said it's only one person even though I have MyChart already and then my (son is a patient at the University of Chicago but they said, "No, there's only one person." It's either me or my husband and I said, "I don't want to change anything," and they'll lose all the information from my son's MyChart so I just let it be. (Patient 30) |
| Technology Training^b^ | In-person training | Healthcare organizations | Maybe have classes or like you said to have help desks for the older generation or people that are not tech-savvy, to be able to help them to teach them how to do it and show them so they're more comfortable with it. (Patient 23)  Host a workshop or once that family member comes with that individual to the appointment, they can have staff on hand to help walk them through how to do it, or show them resources in-person at one of their visits. Sort of like how when you're in the hospital, someone comes around and registers you. There can be someone like that. A social worker. (Patient 10)  I know as for myself, doctors, when you go into the office, you are not the only patient. They can't spend all of their time with you. I feel like they should have an appointment where they have a guy with you and then they will go over some things with you to help you understand it. (Patient 13) |
|  | Remote training | Healthcare organizations; | I guess I'm so used to doing everything on the phone. I think maybe it's because I'm older and it's hard for me to make a change. Sometimes it's hard to relearn something or to learn something when you are so used to doing it this way and this way works for you. (Patient 44)  "They need to have a live person, where you can pick up the telephone and call and say, ‘You know what? I need you to help me troubleshoot. I got a conference, a video call, I don't seem to be able to get in.’ You give them the access codes and things, and they'll be able to see if your access codes are right." (Patient 2) |
|  | Enhance community training resources | Community organizations | It would be good to have a person close in the neighborhood or whatever to be there to help. I don't know how to tell you about that because it's just what I feel. I'm 70 years old, [chuckles] for seniors it's better to have a one-on-one. I believe that. That's it. (Patient 36)  I think that the community where we live at...it's resources that assist with like computer classes that get people used to using a computer or even just to know that normal functions of a computer so there are resources that assist with that...Training about how to, just the basics training. Because we're so social, we're social people, so what is to help one another. Got to start somewhere. Children at school, resource programs and community centers. (Patient 20)  What could change is possibly having places where people are able to access this type of stuff. Even if they do have access to it but they don't know how to use it, then having the resources for them to learn how to use it. (Patient 22) |
|  | Increase training resource awareness | Healthcare organizations; Community organizations | Well, there's nothing like advertising. If you guys wanted to catch up people to speed, you have always those post-it. If you're interested in a research lab or if you're speak up program, all those, you can have another flyer, "Hey, whatever the class may be that you want to, that you think would help out. If you want basic computer these facilities are available, sign up." If a school were willing to do something like that, that would be the place to announce it. I'd definitely look at it. I'd be like, "Wow." (Patient 51) |
|  | Intergenerational support | Senior citizens; Technology limited individuals; Extended family | I experience it on my own because, like I just told you, I have my kids help me with everything for the internet, even for my emails. I don't know how to do any of that. (Patient 54)  There are of course older people that I do know that would not know how to do something without the help of maybe somebody who does know how. I know the older generation aren't so good with technology, I guess we could say. (Patient 29) |
| Support low-tech options | Low-tech communication (e.g., mailing letters, phone calls) | Healthcare organizations | For seniors, I think it would be something is that-- I'm only 53--so I'm a younger person. Probably if they would mail something in the mail Something more physical. (Patient 12) |
|  | Low-tech healthcare delivery (e.g., audio-only phone visits, home visits” | Government; Healthcare organizations | I think if it was via phone, because we do have a house phone, he probably would be able to use that. Obviously, if they would have called him, he would have answered. That would have been okay but for the most part, anything other than that he wouldn't be able to do. He has trouble calling out because he can't see the buttons on his phone. (Patient 48)  Very slow internet. We're in like a dead zone. We've called AT&T. AT&T has not gone and upped our speed. I get so frustrated because my computer is so slow. It's almost dial-up! I was supposed to have a video visit. But it don’t support Zoom. It doesn't have enough of-- What do you call it? Enough gigabytes or-- It doesn't have enough of something to support it. It's a lot of stuff that I can't get because of the speed. I went on the internet. They sent me a code, and I put the code in, and it say, "This computer would not support it." That was frustrating because I tried it several times just to make sure that I wasn't doing something wrong. This time I would put the numbers in that they sent me, it would tell me the same thing, cannot support.”  I called the clinic, and I let them know to let my doctor know that I cannot get Zoom, it would have to be a phone call. I have to do the phone. The next time I talk to my doctor would be on the phone because she's already set it up. I think in three weeks' time, she'll be calling me back. Sometimes it's even better with the phone, you don't have to go out. Between me and my husband, we are at University Chicago at least 10 times a month and that's quite a bit. She has so many appointments. It's nice to be at home and talk to a doctor. You telling them how you feel, what's hurting you. They will tell you what you need to do, "You need to come in for this," it's the same thing like in-person. (Patient 44)  Just being able to not have to drag her out, I wish she can get her COVID shot delivered instead of having to go in for it... because my mom is 95. We're in an apartment complex... a big one. Her apartment's at the end of the hallway from the elevator. I don't even know, we're trying to figure out how we're even going to be able to get her down the hallway because she hadn't been on the hallway in a while. On the phone, doctor's visits are fine. She'll be 96 this year, so it's just going to make it hard for her to do in-person. (Patient 45) |

^a^ The general term “technology” includes both high speed accessible internet access as well as supported devices such as computers, laptops, tablets, and/or smartphones.

^b^ Includes sentiments of general technology training, as well as more focused recommendations to support training specific to healthcare applications such as video visits and patient portals.
